# Beyond Genetics in Glioma Pathways: The Ever-Increasing Crosstalk between Epigenomic and Genomic Events

**DOI:** 10.1155/2012/519807

**Published:** 2012-06-18

**Authors:** Ramón Martínez

**Affiliations:** Department of Neurosurgery, University of Goettingen, Robert-Koch-Straße 40, 37075 Goettingen, Germany

## Abstract

Diffuse gliomas are the most frequent brain tumor in adults. This group of brain neoplasms, ranging from histologically benign to aggressive malignant forms, represents a challenge in modern neurooncology because of the diffuse infiltrative growth pattern and the inherent tendency to relapse as a more malignant tumor. Once the disease achieves the stage of glioblastoma multiforme (GBM), the prognosis of patients is dismal and the median survival time is 15 months. Exhaustive genetic analyses have revealed a variety of deregulated genetic pathways involved in DNA repair, apoptosis, cell migration/adhesion, and cell cycle. Recently, investigation of epigenetic alterations in gliomas has contributed to depict the complexity of the molecular lesions leading to these malignancies. Even though, the efficacy of the state-of-the-art form of chemotherapy in malignant gliomas with temozolomide is based on the methylation-associated silencing of the DNA repair gene *MGMT*. Nevertheless, the whole scenario including global DNA hypomethylation, aberrant promoter hypermethylation, histone modification, chromatin states, and the role of noncoding RNAs in gliomas has only been partially revealed. We discuss the repercussion of epigenetic alterations underlying deregulated molecular pathways in the pathogenesis and evolution of gliomas and their impact on management of patients.

## 1. Introducing the Challenge: The Management of Gliomas 

**Figure 1:**
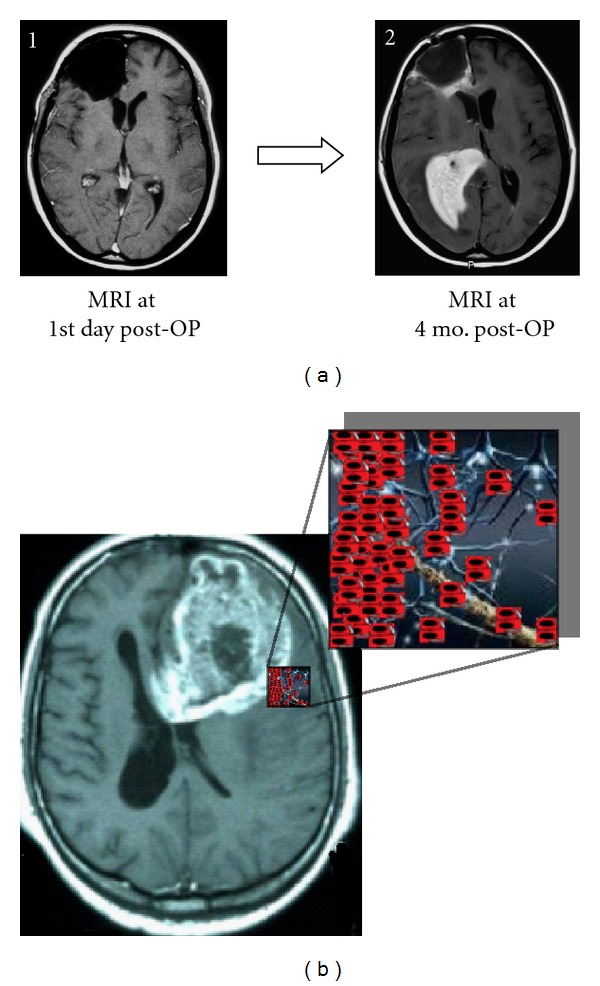
(a)   T1-weighted, gadolinium-enhanced axial MRI showing the resection hole one day after surgical extirpation of a right frontal glioblastoma multiforme (1). Four months after surgery, fractionated radiotherapy (59 Gy), and chemotherapy with temozolomide, a new right periventricular tumor manifestation was observed invading the basal ganglia. An enhancement in the dorsal resection hole is evidenced, also indicating the local relapse (2). MRI gently provided by Professor A. Giese (Department of Neurosurgery, University of Mainz, Germany). (b) T1-weighted, gadolinium-enhanced axial MRI revealing a large left frontal glioblastoma. The illustration depicts the outer tumor border (as defined by the gadolinium enhancement) with glioblastoma cells migrating far away through the brain tissue.

Gliomas are the most frequent primary brain tumors in adults accounting for more than 70% of all brain neoplasms and display a group of tumors with different features regarding morphology, genetic and epigenetic aberrations, and response to therapy [1]. An additional relevant feature of gliomas represents its great tendency to infiltrate into adjacent normal brain tissue. The outer border of gliomas, as observed by T1-weighted magnet resonance imaging (MRI), does not delineate the true dimension of the tumor. Moreover, tumor cells diffusely invade the normal brain and can be detected far beyond rendering this condition incurable by combined surgery, radio, and chemotherapy (Figure 1). This is more true for glioblastoma multiforme (GBM), which accounts for about 60% of all gliomas and 12–15% of all brain tumors, and it is per se the most frequent primary brain tumor [1, 2]. It is one of the most devastating and lethal forms of human cancer despite the significant efforts that have been made to unravel its molecular basis. In Europe and North America, the incidence is three new cases per 100,000 inhabitants per year (Central Brain Tumor Registry of the United States, CBTRUS, http://www.cbtrus.org/). Although GBM can manifest itself at any age, it preferentially occurs in adults, with a wide peak age of incidence between 45 and 70 years [3].

In spite of all the progress in the fields of surgery and radiochemotherapy, including the milestone of chemotherapy with the alkylating drug temozolomide, the prognosis of GBM is still dismal [3–5]. Recently published meta-analyses reported that, despite multimodal therapy with gross total resection and radio- and chemotherapy with temozolomide, an improvement of only few months in median survival time (14.6 months) had been achieved compared to surgery plus radiotherapy alone (12.1 months) [6]. As a general rule, tumor recurrence develops after a short relapse-free period, signaling a survival time of 5–8 months [4]. 

## 2. Crosstalk between Genetics and Epigeneticsin Gliomas

In benign astrocytomas WHO grade II, the most common genetic alteration is mutations of the *TP53* gene in about 60% of the cases as well as mutations of the isocitrate dehydrogenase 1 (*IDH1*) gene in about 70% of the tumors [1]. The rates of these mutations may vary between subtypes, for instance *TP53* mutations have been observed in up to 80% of astrocytomas with gemistocytic features [7]. Epigenetic alterations in grade II astrocytomas are promoter hypermethylation of the DNA repair gene *MGMT*, the protocadherin-gamma subfamily A11 (*PCDH-gamma-A11*) and the tumor suppressor gene *EMP3* [8]. Interestingly in grade II gliomas without *TP53* mutations, epigenetic downregulation of *CDKN2A*, which regulates the *MDM2*-associated p53 degradation, is frequently observed [9]. In anaplastic astrocytomas WHO grade III, *TP53* and *IDH1* mutations have been observed with similar frequencies to grade II tumors. Moreover, mutations of the *RB1* gene occur in about 25% of the cases. Furthermore, the cell-cycle regulator genes *CDKN2A* and *CDKN2B* undergo commonly inactivation via genetic mutation and epigenetic silencing [10].

**Figure 2:**
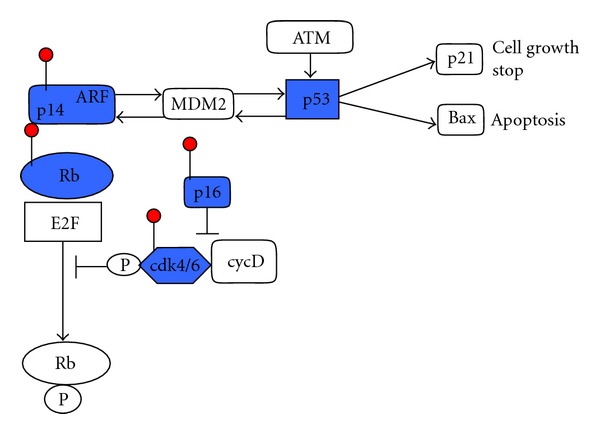
*TP53*/*MDM2*/p14^ARF^ and cell cycle p16^INK4a^/*CDK4*/*RB1* networks and targets of hypermethylation-mediated inactivation (marked by red stocks).

Concerning GBM, two patterns have so far been described in the pathogenesis through the gatekeeper pathway. These have different molecular profiles. Type 1 GBM typically shows inactivation of the *TP53* tumor suppressor gene but no amplification of the *EGFR* oncogene. Mutations of *p53*, mostly associated with loss of heterozygosity (LOH) in the 17p chromosome region, can be observed in GBM originating from a less malignant glioma precursor. *TP53* inactivation does not occur together with amplification of the *EGFR* oncogene, which is only identified in GBM without *TP53 *mutation [1, 11–13]. More than 70% of malignant gliomas show a deregulated *TP53* pathway not only by mutation of *TP53 *but also amplification of *MDM2*, homozygous deletion/mutation, or promoter hypermethylation-mediated silencing of *CDKN2A *as shown in Figure 2 [10, 14].

In contrast, type 2 GBM shows overexpression or amplification of the *EGFR* without mutations of *TP53* [11, 15], and it appears de novo, that is, in patients without a less malignant precursor neoplasm such as grade II or III astrocytoma [16]. The former molecular data strongly pinpoint to two independent GBM pathogenetic pathways [17]. Moreover, *EGFR* amplification is almost always consistent with LOH in chromosome region 10q [16]. The tumor suppressor gene *PTEN,* mapping the 10q23 region, is mutated in approximately 30% of type 2 GBM [18]. Mutations in this gene have been described only in malignant gliomas and are rarely associated with *p53 *mutations. Other frequent mutations in type 2 GBM affect the *CDK* cell-cycle-regulator genes. Amplification of *CDK4 *and *CDK6* was observed in 15% of type 2 GBMs [10]. Mutations of the cell-cycle-regulator genes *CDKN2A/CDKN2B* have been observed in 40% of all GBM. Moreover, a functional loss of expression of the *CDKN2A *gene by promoter hypermethylation was found in 15% of GBM as well [19]. Mutations of the *IDH1 *gene have been frequently observed in those GBM progressing from a less malignant precursor lesion, that is in type 1 GBM, mostly of them affecting young patients. Interestingly, these *IDH1* mutations were associated with a better outcome [20, 21]. 

In addition to type 1 and type 2 GBMs, there are other forms, whose molecular profiles do not identify them as belonging to either of the two classic pathways [12, 15, 22]. A small subgroup of these GBMs is found to harbor LOH on chromosomes 1p and 19q, which have been suspected for a long time of containing tumor suppressor genes [15, 23].

In recent years, the epigenetic pattern of gliomas has received increasingly attention and many efforts have been done to define it. Nevertheless, it is only partially devised. Global DNA hypomethylation in gliomas, affecting up to 10 million CpG dinucleotides per haploid tumor genome, actually occurs in GBM [24]. This phenomenon appears to be associated with malignant evolution of cells through activation of oncogenes, promotion of genomic instability, or the loss of imprinting. Moreover, the significance of hypermethylation of CpG island of gene promoters in GBM is highlighted by the observation of epigenetic-mediated inactivation of a wide variety of genes associated with tumor suppression (*RB1, VHL, EMP3*, *RASSF1A, BLU*), cell cycle regulation (*CDKN2A/CDKN2B*), DNA repair (*MGMT, hMLH1*), and tumor invasion and apoptosis (*DAPK1, TIMP3, CDH1*, the protocadherin family member *PCDH-gamma-A11, TMS1/ASC*) [25–31]. These epigenetic events have been described in oligodendrogliomas and ependymomas as well [32].

The methylation signature of gliomas is also rather associated with tumor lineage and malignancy grade. Thus, astrocytomas grades WHO II and III and GBM grade IV show different methylation status of several genes [33]. Even though, primary and secondary GBMs were found to differ concerning methylation of genes which was associated with decreased mRNA levels [34]. In this context, methylation of *MGMT* is more frequently observed in 75% of secondary GBM than in primary GBMs (36%) (http://www.iarc.fr/p53 and [29]). Moreover, *MGMT* methylation has been observed to be associated with *TP53 *mutations in secondary GBMs. 

## 3. Cancer Epigenetics and Disrupted CellularPathways by Methylation-Associated Events

Epigenetic alterations have been linked with all cancer types. The spectrum of lesions includes gain and loss of DNA methylation including multicopy elements as well as single-copy genes. Most of these aberrations, mainly those followed on DNA methylation and deacetylation of histones, change gene expression and genome stability through regulation of local chromatin structure. Moreover, recent data suggest that early epigenetic changes occur during tumorigenesis and that they may predispose progenitor cells to further molecular changes that are involved in tumor promotion [35, 36]. Given the high frequency of DNA methylation changes, these events may become ideal biomarkers for early molecular diagnostics, such as *MGMT*, which has been observed to be hypermethylated in low-grade gliomas (grade II) further evolving to gliomas grade III and GBM [37]. Furthermore, this biomarker allows neurooncologists to predict patient's response to current chemotherapy with temozolomide [6].

**Figure 3:**
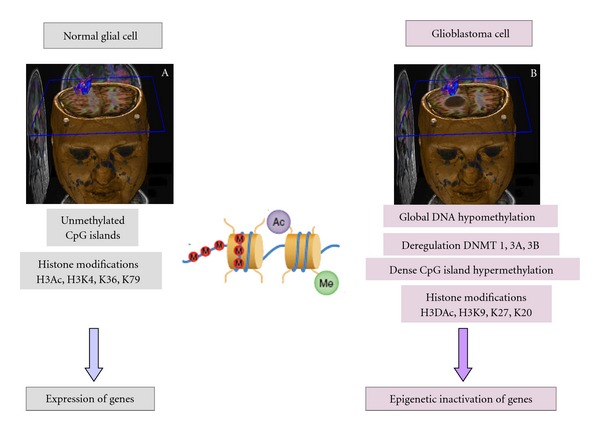
(a) 3D MRI. Normal glial cells CpG islands in the promoter regions of genes lack of methylation, which is a prerequisite for active gene transcription. Fully methylated CpG islands are found only in the promoters of silenced alleles for selected imprinted autosomal genes and silenced genes on the inactivated X-chromosomes of females. (b) 3D MRI showing a right frontal GBM, in which cells change their methylation pattern in a wide manner including deregulation of methylating DNA methyl-transferases 1, 3A, and 3B, global hypomethylation through demethylation in CpG islands of the promoters of a wide variety of genes as well as a severe hypermethylation locally affecting unmethylated regions. Densely methylated DNA is associated with deacetylated histones and compacted chromatin, which is refractory to gene transcription.

Cancer cells change their methylation pattern in a wide-ranging manner. For instance, the regulation of methylating DNA methyltransferases 1, 3A, and 3B is altered profoundly. Moreover, there is a global DNA hypomethylation through demethylation of the promoters of a wide variety of genes as well as a severe hypermethylation that locally affects normally unmethylated regions, mainly CpG islands. As a rule, densely methylated DNA is associated with deacetylated histones and compacted chromatin, which is refractory to gene transcription (Figure 3) [38, 39]. DNA epigenetic alterations without gene mutations have been found to be common events in the pathogenesis of a wide variety of cancers, including gliomas, especially the methylation-associated silencing of tumor suppressor genes such as *VHL, p*16^INK4a^, *E-cadherin, hMLH1, BRCA1*, and *LKB1* [40, 41]. Promoter hypermethylation has been observed in a large number of regulator genes in tumor pathogenesis, including *p*15^INK4b^ (hypermethylated in hematological malignancies), *p73* (hypermethylated in lymphomas) and *ER* (receptor for estrogen-induced transcriptional activation), the DNA repair genes *MGMT *and* GSTP1* (related to the prevention of oxidative DNA damage), *TIMP3* and *DAPK1 *[42–44].

An overview of disrupted cellular pathways in gliomas by hypo/hypermethylation of gene promoters or histone modifications is provided in Table 1.

### 3.1. Mutator Pathways

#### 3.1.1. DNA Mismatch Repair System

Genomic instability plays a fundamental role in the pathogenesis of a wide variety of solid cancers [45, 46]. One form is the chromosomal instability (CIN), characterized by multiple gains and losses at specific chromosome regions [45]. In GBM, CIN has been found to be related with two pathways, typically harboring either mutations of *TP53* or amplification of the *EGFR* oncogene. A second form of genomic instability is the microsatellite instability (MSI), which typically displays length mutations at microsatellite sequences in noncoding and in coding regions of genes such as *PTEN, TGFßR-II, IGFIIR*, and *BAX* [47–50]. MSI is the consequence of the inactivation of genes driving the DNA mismatch repair system [47, 48, 51] and has previously been observed in hereditary syndromes, such as the hereditary nonpolyposis colorectal cancer (HNPCC) with a frequency up to 90% [48, 51]. In these cases, the defect is attributed to germ line mutations in mismatch repair genes, mainly *MLH1* and *MSH2*. However, in sporadic malignancies, MSI without mutations of those genes could be identified in colorectal (15%), nonsmall cell lung (35%), head and neck (28%), gastric (10%), and prostate cancers (8.5%) [52–56]. In GBM, MSI has been detected as well, with frequencies ranging from 5–50% [57]. Remarkably, in these sporadic tumors, MSI was not associated to mutations of the mismatch repair system but to *MLH1* promoter hypermethylation-mediated silencing. Interestingly, MSI was observed to be more frequent in those GBMs evolving from less malignant gliomas grade II or III, which typically display *TP53* mutations without *EGFR* amplification, as well as in relapse GBM [57]. Taking this into consideration, inactivation of tumor suppressor genes may represent the final stage in the malignant transformation of cells which are driven by an increasing genomic instability through the early inactivation of the DNA mismatch repair gene *MLH1.*

#### 3.1.2. *MGMT *DNA Repair Gene

**Figure 4:**
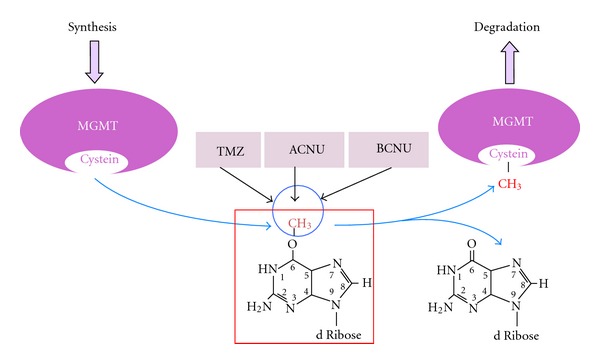
Diagram showing how alkylating drugs temozolomide (TMZ), nimustine (ACNU), and carmustine (BCNU) act damaging DNA by introducing alkyl residues in the O^6^ position of guanine, thus producing DNA interstrand cross-links. The DNA repair O^6^-methylguanine DNA methyltransferase (*MGMT*) reverses the formation of adducts at the O^6^ position of guanine. MGMT transfers the alkyl group from the O^6^-guanine to an active cysteine within its own sequence in a reaction that inactivates one MGMT molecule for each lesion repaired. The alkylated MGMT protein then becomes detached from DNA and is targeted for degradation by ubiquitination.

*MGMT* is another relevant example of a DNA repair regulator gene undergoing methylation-mediated inactivation in gliomas, even in an early phase*.MGMT* hypermethylation has been reported in gliomas grade I to IV from 20 to 90% depending on the histological subtype [58]. It removes mutagenic and cytotoxic adducts from O^6^-guanine in DNA (Figure 4). Alkylation of DNA at the O^6^ position of guanine is a primordial step in tumor formation, primarily due to the tendency of the O^6^-methylguanine to mispair with thymine during DNA replication, leading to the conversion of guanine-cytosine to adenine-thymine pairs in DNA. Furthermore, most common mutations caused by alkylating drugs are G : C to A : T transitions. A functioning *MGMT* provides cells protection against these sequence alterations, by transferring the alkyl group from the O^6^-guanine in DNA to an active cysteine within its own sequence [59]. *MGMT*-silenced cancer cells acquire a mutator phenotype through generation of transition point mutations of genes such as *TP53* or the oncogene *K-ras* [60]. Strikingly, *MGMT* hypermethylation has been observed to be associated with *TP53 *mutations in secondary GBMs. Notably, these *TP53* mutations were almost exclusively G : C to A : T transition mutations and of those, most of them were at CpG sites (http://www.iarc.fr/p53 and [29]).

A link was observed in GBM between *MGMT* promoter methylation and a hypermutator phenotype as a consequence of a mismatch repair deficiency in treated glioblastomas, a finding with potentially significant clinical implications [61].

#### 3.1.3. Chromosomal Instability through Inactivation of *RECQL2* Helicase (*WRN* Gene)

 Mutations in the Werner syndrome (WS) gene (*WRN*) are found in patients exhibiting the clinical features of this rare autosomal recessive disease, displaying premature onset of age-related pathologies [62]. Most of *WRN* mutations lead to loss of function of the protein [62]. The *WRN* gene belongs to the RecQ family of DNA helicases, whose mutations lead to defects in DNA replication, recombination, and repair. Since patients with *WRN* gene germ line mutations develop a wide variety of epithelial and mesenchymal tumors, a tumor suppressor function for *WRN* is anticipated and it is supported by the very frequent loss of heterozygosity at the chromosomal *WRN* region, 8p11.2–p12 in different cancers [63]. Nevertheless, somatic mutations of *WRN* gene have not been found in sporadic malignancies, but epigenetic-mediated silencing was reported in many tumor types of epithelial and mesenchymal lineage in a parallel way observed in *MLH1* [64]. The hypothesis defending the link between the methylation-mediated inactivation of *WRN* and a mutator phenotype in human malignancies is supported by the observation that *WRN* defective cancer cells through methylation are very sensitive to the action of DNA-damaging agents [64]. The significance of methylation of *WRN* in gliomas of different lineages and malignancy grades is a topic which remains to be definitively delineated and might have implications in survival prediction or response to chemotherapy.

### 3.2. Apoptosis

Current anticancer therapy is directed to activate the mitochondria-dependent or intrinsic apoptotic pathway, which will be stimulated by DNA damage induced by radiotherapy or drugs [65]. The success rate of this treatment modality in GBM, with very few exceptions, is actually low. This therapy failure may be in part explained by the apoptosis-resistant phenotype of GBM, an extended hallmark in cancer [66].

Epigenetic inactivation of apoptosis-related genes has been consistently reported in gliomas, such as *TMS1/ASC* and *DAPK1* [67, 68], *WIF-1* [69], *SFRP1* [70]. and *CASP8 *[71], Previously. we have observed an epigenetic silencing of the proapoptotic gene *CASP8 *during progression of primary-to-recurrent GBM [71]. *CASP8 *is also hypermethylated in 40% of neuroblastomas and medulloblastomas, and a high concordance between gene methylation and protein expression was observed as well [72, 73]. *CASP8* encodes a protein at the top of the mitochondria-independent apoptosis cascade in a pathway triggered by ligation of death receptors, such as Fas and tumor necrosis factor-related apoptosis-inducing ligand (TRAIL) death receptors (DR4 and DR5), by their cognate ligands [74, 75]. Once activated *CASP8*, the executioner *CASP3* becomes activated as well, which leads to dismantling of the cell. *CASP8* can also promote the activation of downstream caspases through proteolysis of BID (BH3-interacting domain death agonist protein) [76]. TRAIL (like tumor necrosis factor, TNF, and Fas ligand) is a potent inducer of apoptosis, and it is counteracted by the decoy receptors DcR-1 and 2. Because of the preferential expression of these antagonistic receptors in normal tissues, it was postulated that both protect from TRAIL-induced apoptosis [77]. Taking this into consideration, the main epigenetic deregulation point of the extrinsic apoptosis cascade in gliomas probably involves *CASP8 *and the death receptors. 

### 3.3. *TP53* and Cell Cycle Networks

The *TP53* gene encodes a protein displaying key roles in different substantial processes such as cell cycle, response to DNA damage, cell cycle, cell differentiation, and cell death [78]. Not only the function of *TP53* may result altered by mutations, but also it may result from alterations in *MDM2*,* MDM4*, or *CDKN2A*. At least two isoforms of the protein *MDM2 * may interact with *TP53*, and it binds to wild-type and mutant *TP53*, thus inhibiting the ability of wild-type *TP53* to activate transcription from promoter sequences. In normal cells, there is a regulatory feed-back loop since the transcription of *MDM2* can be induced by wild-type *TP53*. In this way, the activity of *TP53* and the expression of *MDM2 *are closely related. Inactivation of *TP53 *in gliomas occurs preferentially in low-grade astrocytomas and secondary glioblastomas (about 65%) [1]. But inactivation of *TP53* without mutations is also reached by overexpression of *MDM2*. Another relevant player in *TP53 *pathway is 14^ARF^, which binds to *MDM2* and inhibits *MDM2*-mediated *TP53* degradation. Hereby, there also is a feed-back regulatory loop since *p*14^ARF^ is negatively regulated by *TP53.* Hypermethylation of *CDKN2A* has been frequently observed in low-grade astrocytomas and secondary GBM in about 30% [79] but also in primary GBM in about 50% of the cases. In this regard, the TCGA project has observed alterations in the *TP53/MDM2*/*CDKN2A* pathway in about 87% of investigated GBM [61].

The cell cycle pathway *CDK4/CDKN2A/RB1* has been found to be disrupted in about 78% of GBM analyzed [61]. The RB1 protein executes the check point G1 into the S phase of the cell cycle. The *CDK4*/cyclin D1 complex induces the activation of the transcription factor E2F through phosphorylation of the RB1 protein, thus activating genes implicated in the cell cycle step G1 → S (Figure 2). On the other hand, *CDKN2A *inhibits the *CDK4*/cyclin D1 complex and, in this way, the transition G1 → S. Hypermethylation-mediated silencing of *RB1* and *CDKN2A *has been frequently observed in primary glioma tissues [80, 81]. 

### 3.4. Noncoding RNAs

MicroRNAs (miRNAs) are small, 20–22-nucleotide noncoding RNAs that have been observed to be encoded by the genome of most of the eukaryotes examined so far. These small molecules have rapidly taken center stage as regulators of gene expression. Recent studies have identified important roles for miRNAs in the development of human cancers; about 200 of them have been associated with the lineage and differentiation of tumor cells, which implies that miRNA levels are crucial to tumor development [82]. Clusters of miRNAs have the properties of classic oncogenes and modulate and are modulated by the activities of other oncogenes [83].

In general, DNA hypomethylation induces a release of miRNA silencing in cancer cells as previously described [84]. One of the miRNA targets is miRNA-124a, which undergoes transcriptional inactivation by CpG island methylation in different human malignancies, in a similar way to that observed with tumor suppressor genes; in particular, a link was observed between the epigenetically mediated loss of function of miRNA-124a with the activation of the oncogenic factor cyclin D kinase 6 as well as the phosphorylation of the tumor suppressor gene* RB1*.

In GBM, molecular analyses have shown that miR-21 has the greatest differential elevated expression in human GBM biopsies, in primary cultures and GBM cell lines compared to those in nonneoplastic fetal and adult brain tissues [85, 86]. Targets implicated in the pathway of miR-21 include *TP53*,* TGF-ß*, the mitochondrial apoptotic pathway, and the tumor suppressor gene *PTEN* [87]. Moreover, knockdown of miR-21 in cultured GBM cells led to increased apoptotic cell death [85]. These findings pinpointed that aberrantly expressed miR-21 contributes to the malignant phenotype by inhibiting the expression of genes involved in apoptosis. Furthermore, miR-21 regulates different genes associated with migration and invasiveness in gliomas, including *RECK*, *TIMP3*, and inhibitors of matrix metalloproteinase. In contrast, downregulation of miR-21 in glioma cells leads to a reduction in their migratory and invasive capability [88]. 

The complexity of this scenario in GBM is further underscored by findings showing a very low expression of other miRNAs such as miR-124, miR-137, miR-7, and miR-128 [89, 90]. Furthermore, transfection of miRNA-124 or miRNA-137 also induced G1 cell cycle arrest in GBM cell lines, which was associated with decreased expression of cyclin-dependent kinase 6 and phosphorylated retinoblastoma proteins [90]. 

Altogether, increasing evidence exists that miRNAs regulate multiple pathways involved in GBM pathogenesis. For most of them, the mechanisms by which miRNAs become altered in GBM are only partially explained. Nevertheless, general regulatory mechanisms (hypermethylation, mutation, deletion, and amplification) are likely to be represented, as well as miRNA-specific mechanisms, for instance altered processing or degradation. But if we keep in mind that protein-coding genes represent about 2% of the whole genome and that noncoding genome regions probably are of unrecognised great importance for physiological tissue development and for disease conditions [91], it is easy to argue that other noncoding RNAs, for instance, transcribed ultraconserved regions, small nucleolar RNAs, PIWI-interacting RNAs, large intergenic noncoding RNAs (lincRNAs) and the group of long noncoding RNAs, might also contribute to the development of human cancers [92]. It is anticipated that this total will become much higher in the near future as further key miRNA/target pairs are experimentally validated. The identification of these miRNAs and their methylation-induced inactivation will create opportunities for new therapeutic strategies by modifying chemoresistance or by inducing sensitivity to radiotherapy.

## 4. Epigenomics: The New Venue in GliomaPathogenesis

Summarizing the reviewed issues, hypermethylation of CpG islands gene promoters is only the tip of the iceberg in the complex context of epigenetic mechanisms contributing to GBM onset. Unraveling the epigenetic lesions in malignant gliomas will open up possibilities for discovering new biomarkers for brain tumor detection and prognosis, as reached with prostate cancer. Restoring epigenetically altered pathways will probably lead to the development of new therapeutic tools with translational significance. We are entering into a new time of personalized molecular treatment, which will allow a successful crosstalk between the lab bench and patient bed, based on a better understanding of genetic and epigenetic mechanisms of human cancers.

## Figures and Tables

**Table 1:** Cellular pathways deregulated in gliomas and associated epigenetic events through promoter hypermethylation, CpGs hypomethylation, and histone alterations leading to modified chromatin states.

Cellular pathway	Genes in gliomas deregulated by hypo/hypermethylation/histone modification
Ras signaling	RASSF1A, RRP22, DIRAS3
Cell migration and adherence	NECL1, E-cadherin, SLIT2, EMP3, TIMP3
Wnt signaling	WIF1, FZD9, IGFBP-3, SFRP family, PEG3
Tyrosine kinase pathways	KIT, SYK, c-ROS
Transcription factors	SOX2, KLF4, GATA 6, ATOH1
Homeobox genes	HOXA 9, HOXA10, HOXA11
Sonic hedgehog signaling	PTCH1, Cyclin D2, Plakoglobin, PAX6, NKX2.2
Notch signalling	NEURL1, HES1, HEY1
BMP developmental pathway	BMPR1B
Hypermutator pathways	hMLH1, hPMS2, MGMT, WRN
Apoptosis	TMS1, DAPK1, CASP8, DR4, DR5
TP53/cell cycle	HIC-1, CDKN2A, RB1, p16^INK4a^
MicroRNAs	miR-124a, miR-21, miR-7, miR-137, miR128

## References

[1] Ohgaki H, Kleihues P (2007). Genetic pathways to primary and secondary glioblastoma. *American Journal of Pathology*.

[2] Ohgaki H, Kleihues P (2005). Population-based studies on incidence, survival rates, and genetic alterations in astrocytic and oligodendroglial gliomas. *Journal of Neuropathology and Experimental Neurology*.

[3] Aldape KD, Okcu MF, Bondy ML, Wrensch M (2003). Molecular epidemiology of glioblastoma. *Cancer Journal*.

[4] Brandes AA, Ermani M, Basso U (2001). Temozolomide as a second-line systemic regimen in recurrent high-grade glioma: a phase II study. *Annals of Oncology*.

[5] Stummer W, Pichlmeier U, Meinel T, Wiestler OD, Zanella F, Reulen HJ (2006). Fluorescence-guided surgery with 5-aminolevulinic acid for resection of malignant glioma: a randomised controlled multicentre phase III trial. *The Lancet Oncology*.

[6] Gorlia T, van den Bent MJ, Hegi ME (2008). Nomograms for predicting survival of patients with newly diagnosed glioblastoma: prognostic factor analysis of EORTC and NCIC trial 26981-22981/CE.3. *The Lancet Oncology*.

[7] Watanabe K, Tachibana O, Sato K, Yonekawa Y, Kleihues P, Ohgaki H (1996). Overexpression of the EGF receptor and p53 mutations are mutually exclusive in the evolution of primary and secondary glioblastomas. *Brain Pathology*.

[8] Riemenschneider MJ, Jeuken JWM, Wesseling P, Reifenberger G (2010). Molecular diagnostics of gliomas: state of the art. *Acta Neuropathologica*.

[9] Watanabe T, Katayama Y, Yoshino A (2007). Aberrant hypermethylation of p14ARF and O6-methylguanine-DNA methyltransferase genes in astrocytoma progression. *Brain Pathology*.

[10] Ichimura K, Bolin MB, Goike HM, Schmidt EE, Moshref A, Collins VP (2000). Deregulation of the p14(ARF)/MDM2/p53 pathway is a prerequisite for human astrocytic gliomas with G1-S transition control gene abnormalities. *Cancer Research*.

[11] Collins VP (2002). Cellular mechanisms targeted during astrocytoma progression. *Cancer Letters*.

[12] Louis DN (1997). A molecular genetic model of astrocytoma histopathology. *Brain Pathology*.

[13] Ohgaki H, Dessen P, Jourde B (2004). Genetic pathways to glioblastoma: a population-based study. *Cancer Research*.

[14] Riemenschneider MJ, Büschges R, Wolter M (1999). Amplification and overexpression of the MDM4 (MDMX) gene from 1q32 in a subset of malignant gliomas without TP53 mutation or MDM2 amplification. *Cancer Research*.

[15] Benjamin R, Capparella J, Brown A (2003). Classification of glioblastoma multiforme in adults by molecular genetics. *Cancer Journal*.

[16] von Deimling A, Louis DN, von Ammon K (1992). Association of epidermal growth factor receptor gene amplification with loss of chromosome 10 in human glioblastoma multiforme. *Journal of Neurosurgery*.

[17] von Deimling A, Louis DN, Wiestler OD (1995). Molecular pathways in the formation of gliomas. *Glia*.

[18] Boström J, Cobbers JM, Wolter M (1998). Mutation of the PTEN (MMAC1) tumor suppressor gene in a subset of glioblastomas but not in meningiomas with loss of chromosome arm 10q. *Cancer Research*.

[19] Nakamura M, Watanabe T, Klangby U (2001). p14ARF deletion and methylation in genetic pathways to glioblastomas. *Brain Pathology*.

[20] Parsons DW, Jones S, Zhang X (2008). An integrated genomic analysis of human glioblastoma multiforme. *Science*.

[21] Yan H, Parsons DW, Jin G (2009). IDH1 and IDH2 mutations in gliomas. *The New England Journal of Medicine*.

[22] Lang FF, Miller DC, Koslow M, Newcomb EW (1994). Pathways leading to glioblastoma multiforme: a molecular analysis of genetic alterations in 65 astrocytic tumors. *Journal of Neurosurgery*.

[23] Kraus JA, Wenghoefer M, Schmidt MC (2000). Long-term survival of glioblastoma multiforme: importance of histopathological reevaluation. *Journal of Neurology*.

[24] Cadieux B, Ching TT, VandenBerg SR, Costello JF (2006). Genome-wide hypomethylation in human glioblastomas associated with specific copy number alteration, methylenetetrahydrofolate reductase allele status, and increased proliferation. *Cancer Research*.

[25] Esteller M, Corn PG, Baylin SB, Herman JG (2001). A gene hypermethylation profile of human cancer. *Cancer Research*.

[26] Waha A, Güntner S, Huang THM (2005). Epigenetic silencing of the protocadherin family member PCDH-*γ*-A11 in astrocytomas. *Neoplasia*.

[27] Horiguchi K, Tomizawa Y, Tosaka M (2003). Epigenetic inactivation of RASSF1A candidate tumor suppressor gene at 3p21.3 in brain tumors. *Oncogene*.

[28] Stone AR, Bobo W, Brat DJ, Devi NS, Van Meir EG, Vertino PM (2004). Aberrant methylation and down-regulation of TMS1/ASC in human glioblastoma. *American Journal of Pathology*.

[29] Nakamura M, Watanabe T, Yonekawa Y, Kleihues P, Ohgaki H (2001). Promoter methylation of the DNA repair gene MGMT in astrocytomas is frequently associated with G:C → A:T mutations of the TP53 tumor suppressor gene. *Carcinogenesis*.

[30] Alaminos M, Dávalos V, Ropero S (2005). EMP3, a myelin-related gene located in the critical 19q13.3 region, is epigenetically silenced and exhibits features of a candidate tumor suppressor in glioma and neuroblastoma. *Cancer Research*.

[31] Tews B, Roerig P, Hartmann C (2007). Hypermethylation and transcriptional downregulation of the CITED4 gene at 1p34.2 in oligodendroglial tumours with allelic losses on 1p and 19q. *Oncogene*.

[32] Alonso ME, Bello MJ, Gonzalez-Gomez P (2003). Aberrant promoter methylation of multiple genes in oligodendrogliomas and ependymomas. *Cancer Genetics and Cytogenetics*.

[33] Uhlmann K, Rohde K, Zeller C (2003). Distinct methylation profiles of glioma subtypes. *International Journal of Cancer*.

[34] Tepel M, Roerig P, Wolter M (2008). Frequent promoter hypermethylation and transcriptional downregulation of the NDRG2 gene at 14q11.2 in primary glioblastoma. *International Journal of Cancer*.

[35] Widschwendter M, Fiegl H, Egle D (2007). Epigenetic stem cell signature in cancer. *Nature Genetics*.

[36] Zhao XD, Han X, Chew JL (2007). Whole-genome mapping of histone H3 Lys4 and 27 trimethylations reveals distinct genomic compartments in human embryonic stem cells. *Cell Stem Cell*.

[37] Brena RM, Plass C, Costello JF (2006). Mining methylation for early detection of common cancers. *PLoS Medicine*.

[38] Jones PA, Baylin SB (2007). The epigenomics of cancer. *Cell*.

[39] Esteller M (2007). Epigenetic gene silencing in cancer: the DNA hypermethylome. *Human Molecular Genetics*.

[40] Esteller M (2002). CpG island hypermethylation and tumor suppressor genes: a booming present, a brighter future. *Oncogene*.

[41] Jones PA, Baylin SB (2002). The fundamental role of epigenetic events in cancer. *Nature Reviews Genetics*.

[42] Bachman KE, Herman JG, Corn PG (1999). Methylation-associated silencing of the tissue inhibitor of metalloproteinase-3 gene suggests a suppressor role in kidney, brain, and other human cancers. *Cancer Research*.

[43] Herman JG, Latif F, Weng Y (1994). Silencing of the VHL tumor-suppressor gene by DNA methylation in renal carcinoma. *Proceedings of the National Academy of Sciences of the United States of America*.

[44] Herman JG, Merlo A, Mao L (1995). Inactivation of the CDKN2/p16/MTS1 gene is frequently associated with aberrant DNA methylation in all common human cancers. *Cancer Research*.

[45] Lengauer C, Kinzler KW, Vogelstein B (1998). Genetic instabilities in human cancers. *Nature*.

[46] Kinzler KW, Vogelstein B (1997). Cancer-susceptibility genes. Gatekeepers and caretakers. *Nature*.

[47] Thibodeau SN, Bren G, Schaid D (1993). Microsatellite instability in cancer of the proximal colon. *Science*.

[48] Ionov Y, Peinado MA, Malkhosyan S, Shibata D, Perucho M (1993). Ubiquitous somatic mutations in simple repeated sequences reveal a new mechanism for colonic carcinogenesis. *Nature*.

[49] Rampino N, Yamamoto H, Ionov Y (1997). Somatic frameshift mutations in the BAX gene in colon cancers of the microsatellite mutator phenotype. *Science*.

[50] Kong D, Suzuki A, Zou TT (1997). PTEN1 is frequently mutated in primary endometrial carcinomas. *Nature Genetics*.

[51] Aaltonen LA, Peltomaki P, Leach FS (1993). Clues to the pathogenesis of familial colorectal cancer. *Science*.

[52] Lothe RA, Peltomaki P, Meling GI (1993). Genomic instability in colorectal cancer: relationship to clinicopathological variables and family history. *Cancer Research*.

[53] Renault B, Calistri D, Buonsanti G, Nanni O, Amadori D, Ranzani GN (1996). Microsatellite instability and mutations of p53 and TGF-*β* RII genes in gastric cancer. *Human Genetics*.

[54] Field JK, Kiaris H, Howard P, Vaughan ED, Spandidos DA, Jones AS (1995). Microsatellite instability in squamous cell carcinoma of the head and neck. *British Journal of Cancer*.

[55] Wooster R, Cleton-Jansen AM, Collins N (1994). Instability of short tandem repeats (microsatellites) in human cancers. *Nature Genetics*.

[56] Watanabe M, Imai H, Kato H (1996). Microsatellite instability in latent prostate cancers. *International Journal of Cancer*.

[57] Martinez R, Schackert HK, Appelt H, Plaschke J, Baretton G, Schackert G (2005). Low-level microsatellite instability phenotype in sporadic glioblastoma multiforme. *Journal of Cancer Research and Clinical Oncology*.

[58] Hegi ME, Diserens AC, Gorlia T (2005). MGMT gene silencing and benefit from temozolomide in glioblastoma. *The New England Journal of Medicine*.

[59] Pegg AE, Dolan ME, Moschel RC (1995). Structure, function, and inhibition of O6-alkylguanine-DNA alkyltransferase. *Progress in Nucleic Acid Research and Molecular Biology*.

[60] Esteller M, Herman JG (2004). Generating mutations but providing chemosensitivity: the role of O 6-methylguanine DNA methyltransferase in human cancer. *Oncogene*.

[61] Cancer Genome Atlas Research Network (2008). Comprehensive genomic characterization defines human glioblastoma genes and core pathways. *Nature*.

[62] Friedrich K, Lee L, Leistritz DF (2010). WRN mutations in Werner syndrome patients: genomic rearrangements, unusual intronic mutations and ethnic-specific alterations. *Human Genetics*.

[63] Nakayama H (2002). RecQ family helicases: roles as tumor suppressor proteins. *Oncogene*.

[64] Agrelo R, Cheng WH, Setien F (2006). Epigenetic inactivation of the premature aging Werner syndrome gene in human cancer. *Proceedings of the National Academy of Sciences of the United States of America*.

[65] Norbury CJ, Zhivotovsky B (2004). DNA damage-induced apoptosis. *Oncogene*.

[66] Hanahan D, Weinberg RA (2000). The hallmarks of cancer. *Cell*.

[67] Stone AR, Bobo W, Brat DJ, Devi NS, Van Meir EG, Vertino PM (2004). Aberrant methylation and down-regulation of TMS1/ASC in human glioblastoma. *American Journal of Pathology*.

[68] Martinez R, Schackert G, Esteller M (2007). Hypermethylation of the proapoptotic gene TMS1/ASC: prognostic importance in glioblastoma multiforme. *Journal of Neuro-Oncology*.

[69] Yang Z, Wang Y, Fang J (2010). Downregulation of WIF-1 by hypermethylation in astrocytomas. *Acta Biochimica et Biophysica Sinica*.

[70] Martinez R, Martin-Subero JI, Rohde V (2009). A microarray-based DNA methylation study of glioblastoma multiforme. *Epigenetics*.

[71] Martinez R, Setien F, Voelter C (2007). CpG island promoter hypermethylation of the pro-apoptotic gene caspase-8 is a common hallmark of relapsed glioblastoma multiforme. *Carcinogenesis*.

[72] Teitz T, Wei T, Valentine MB (2000). Caspase 8 is deleted or silenced preferentially in childhood neuroblastomas with amplification of MYCN. *Nature Medicine*.

[73] Zuzak TJ, Steinhoff DF, Sutton LN, Phillips PC, Eggert A, Grotzer MA (2002). Loss of caspase-8 mRNA expression is common in childhood primitive neuroectodermal brain tumour/medulloblastoma. *European Journal of Cancer*.

[74] Rehemtulla A, Hamilton CA, Chinnaiyan AM, Dixit VM (1997). Ultraviolet radiation-induced apoptosis is mediated by activation of CD- 95 (Fas/APO-1). *Journal of Biological Chemistry*.

[75] Thorburn A (2004). Death receptor-induced cell killing. *Cellular Signalling*.

[76] Wang X (2001). The expanding role of mitochondria in apoptosis. *Genes and Development*.

[77] Pan G, Ni J, Wei YF, Yu GL, Goodwin R, Dixit VM (1997). An antagonist decoy receptor and a death domain-containing receptor for TRAIL. *Science*.

[78] Bögler O, Huang HJ, Kleihues P, Cavenee WK (1995). The p53 gene and its role in human brain tumors. *Glia*.

[79] Nakamura M, Watanabe T, Klangby U (2001). p14ARF deletion and methylation in genetic pathways to glioblastomas. *Brain Pathology*.

[80] Nakamura M, Yonekawa Y, Kleihues P, Ohgaki H (2001). Promoter hypermethylation of the RB1 gene in glioblastomas. *Laboratory Investigation*.

[81] Wolter M, Reifenberger J, Blaschke B (2001). Oligodendroglial tumors frequently demonstrate hypermethylation of the CDKN2A (MTS1, p16INK4a), p14ARF, and CDKN2B (MTS2, p15INK4b) tumor suppressor genes. *Journal of Neuropathology and Experimental Neurology*.

[82] Lu J, Getz G, Miska EA (2005). MicroRNA expression profiles classify human cancers. *Nature*.

[83] He L, Thomson JM, Hemann MT (2005). A microRNA polycistron as a potential human oncogene. *Nature*.

[84] Lujambio A, Esteller M (2009). How epigenetics can explain human metastasis: a new role for microRNAs. *Cell Cycle*.

[85] Chan JA, Krichevsky AM, Kosik KS (2005). MicroRNA-21 is an antiapoptotic factor in human glioblastoma cells. *Cancer Research*.

[86] Ciafrè SA, Galardi S, Mangiola A (2005). Extensive modulation of a set of microRNAs in primary glioblastoma. *Biochemical and Biophysical Research Communications*.

[87] Papagiannakopoulos T, Shapiro A, Kosik KS (2008). MicroRNA-21 targets a network of key tumor-suppressive pathways in glioblastoma cells. *Cancer Research*.

[88] Gabriely G, Wurdinger T, Kesari S (2008). MicroRNA 21 promotes glioma invasion by targeting matrix metalloproteinase regulators. *Molecular and Cellular Biology*.

[89] Godlewski J, Nowicki MO, Bronisz A (2008). Targeting of the Bmi-1 oncogene/stem cell renewal factor by MicroRNA-128 inhibits glioma proliferation and self-renewal. *Cancer Research*.

[90] Silber J, Lim DA, Petritsch C (2008). miR-124 and miR-137 inhibit proliferation of glioblastoma multiforme cells and induce differentiation of brain tumor stem cells. *BMC Medicine*.

[91] Mercer TR, Dinger ME, Mattick JS (2009). Long non-coding RNAs: insights into functions. *Nature Reviews Genetics*.

[92] Esteller M (2011). Non-coding RNAs in human disease. *Nature Reviews Genetics*.

